# Prevalence of infectious diseases and its associated factors among the blood donors of the Honduran Red Cross – Northern Region between 2014 and 2016

**DOI:** 10.1371/journal.pone.0207338

**Published:** 2018-11-15

**Authors:** Gustavo Hernández-Arriaga, Karen Ruglas, César Alas-Pineda, Carmen Chinchilla-López, Glenda Arriaga-Mendoza, Suyapa Bejarano-Cáceres, Christian R. Mejía

**Affiliations:** 1 Facultad de Medicina y Cirugía, Universidad Católica de Honduras—Campus San Pedro y San Pablo, San Pedro Sula, Cortés, Honduras; 2 ASOCEM Universidad Católica de Honduras–San Pedro y San Pablo (ASOCEM UNICAH–SPSP), San Pedro Sula, Cortés, Honduras; 3 Cruz Roja Hondureña, San Pedro Sula, Cortés, Honduras; 4 Liga Contra el Cáncer de Honduras, San Pedro Sula, Cortés, Honduras; 5 Escuela de Medicina Humana, Universidad Continental, Huancayo, Perú; University of Cincinnati College of Medicine, UNITED STATES

## Abstract

**Introduction:**

A number of parenteral infections in third-world countries are caused by blood transfusions. Our objective was to determine the prevalence of and factors associated with infected blood obtained by the Honduran Red Cross through blood donations, to ensure the safety of the donated blood.

**Materials and methods:**

This study used a cross-sectional analytical design based on the secondary analysis of data. Information on blood donors from San Pedro Sula, Honduras, between 2014 and 2016 were obtained from the database of the Honduran Red Cross. Data analysis was performed in two phases. The first phase described the variables, with the values presented as frequencies and percentages for categorical variables. The second phase involved a statistical analysis using generalized linear models.

**Results:**

The proportions of donors who tested positive for syphilis, core hepatitis, hepatitis B, human T-cell lymphotropic virus, human immunodeficiency virus, and hepatitis C infections were 45% (n = 447), 35% (348), 11% (105), 10% (97), 6% (59), and 3% (24), respectively. The results of multivariate analysis demonstrated that the number of women positive for HIV infection was lower than that of men (p = 0.006). Older participants were more likely to be positive for core hepatitis (p = 0.029) and syphilis (p<0.001) infection but less likely to be positive for hepatitis B (p<0.001), hepatitis C (p = 0.027), human immunodeficiency virus (p<0.001), and human T-cell lymphotropic virus (p<0.001) infection compared to younger participants. Replacement donors had an increased likelihood of positivity for core hepatitis (p = 0.003) infections but a decreased likelihood of positivity for human T-cell lymphotropic virus infection (p = 0.001).

**Discussion:**

The high prevalence of infectious diseases in Honduras warrants the need for monitoring donated blood to prevent infected blood from being provided for transfusions. Furthermore, education efforts through the creation of prevention programs are necessary to educate the Honduran population, especially younger individuals, about transfusion-transmissible infections.

## Introduction

The Honduran Red Cross is a humanitarian organization that has been recognized by the government since 1937. This organization is composed of national relief society volunteers, who serve as independent, autonomous auxiliaries of public authorities and form a part of the military health service, in accordance with the provisions of the Geneva Conventions [1]. Additionally, this organization follows international standards to ensure the safety of donated blood and other components [2]. Considering that multiple morbidities resulting from inappropriate blood donations have been reported [3, 4], studies on the prevalence of infectious diseases related to blood transfusion and its associated factors among the blood donors of the Honduran Red Cross should be conducted, particularly in the northern region, because this organization is responsible for collecting the highest number of donations nationwide and is the leading organization that supplies blood to hospitals [5].

In developing countries, the relative risk of donation is heterogeneous due to geographical diversity, habitat, and population groups; in this context, 1 in every 10 000 blood donations is infected with human immunodeficiency virus (HIV), and 2–3 in every ten persons who received blood transfusions were infected with hepatitis B virus (HBV) and hepatitis C virus (HCV) [6]. Additionally, blood-borne diseases, such as HIV (0.15%), HBV (0.28%), HCV (0.35%), and human T-cell lymphotropic virus types I and II (HTLV-I and HTLV-II) (0.14%) infections and syphilis (1.01%), are prevalent in Honduras [7]. Moreover, other studies have also reported transfusion-transmissible infectious diseases among blood donors [8,9]. Hence, monitoring donated blood is important to ensure the safety of the donated blood.

Therefore, this study determined the prevalence of infectious diseases and the associated factors among blood donors in the northern region of the Honduran Red Cross between 2014 and 2016.

## Materials and methods

This study used a cross-sectional analytical design based on a secondary analysis of data. The data were obtained from the database of the Honduran Red Cross, which had information on blood donors from San Pedro Sula, Honduras, between 2014 and 2016. This project was approved by the Institutional Ethics Committee of the “Universidad Católica de Honduras.” The Honduran Red Cross is a major blood center in Honduras [10] and serves a population of over one million residents. [11]. This study included donors who tested positive for at least one of the evaluated infectious diseases. Eight infection-positive donors were excluded from the statistical analysis due to missing variables such as age, civil status, scholarship, and type of donation (accounting for 70% of the total information).

A minimum difference of 5% was calculated to determine the minimum sample size of the donors with a 95% confidence level and 85% statistical power within a single population. This calculation yielded a minimum sample size of 896 people, with an additional 10% to compensate for possible losses. Hence, data from at least 986 donors was required. Considering that period reports were evaluated in this study, a census-type sampling was used.

Sample Collection: Blood samples from each subject were collected aseptically using sterile needles, placed into sterile serum collection bottles, and centrifuged at 1,500 rpm for 15 minutes. The separated serum was placed in sterile bottles and stored at 2°C.

The types of infectious diseases/conditions of the donors, the main variables evaluated in this study, were grouped into six categories: core hepatitis, HBV, HCV, HIV, syphilis, and HTLV infections. The diagnostic process was performed by the Honduran Red Cross–northern region team to identify donors positive for infectious markers at the time of donation. Internationally-approved diagnostic reagents were used in this study. Additionally, samples from donors enrolled in this study were tested for markers of HCV, HBV, HIV, hepatitis core, HTLV and syphilis infections by chemiluminescent microparticle immunoassays (CIMAs) (Architect, Abbott, USA). The presence of HCV was tested in duplicate samples based on the qualitative detection of antibodies to HCV (anti-HCV) in human serum and plasma. HIV p24 antigen and antibodies to HIV I-II were simultaneously and qualitatively detected in human serum or plasma. The presence of HBV surface antigenemia (HBsAg) indicated that the donor was in the infectious stage, while the detection of antibodies to HBV core (anti-HBc) was indicative of a previous or ongoing HBV infection within an undefined time. Antibodies to HTLV were screened by the qualitative detection of antibodies to HTLV-I and HTLV-II in human serum and plasma. The syphilis assay provided the qualitative detection of immunoglobulin G and M (IgG and IgM) antibodies against *Treponema pallidum* (TP) in human serum and plasma.

The associated social and educational variables included donor age (treated as quantitative data), sex (male or female), marital status (single, married, or other), educational level (none or primary, secondary, or tertiary level), history of previous donation (yes or no), and type of blood donor (voluntary or replacement). Furthermore, the year in which the donation was made was also included in the analysis.

Following the approval of the study protocol, data collection was carried out by the authors of the present study. A data collection form was created using Microsoft Excel (version 2013 for Windows; Microsoft, Redmond, WA, USA) to ensure orderly and meticulous extraction of the information. The statistical author was responsible for data depuration and quality control. All data were transferred to a Stata worksheet (version 11.1; StataCorp LLC, College Station, TX, USA).

The data analysis was carried out in two phases. In the first phase, the variables were described, with the values presented as frequencies and percentages for categorical variables. Furthermore, the median age of the participants was also obtained because they are not typically evaluated using the Shapiro–Wilk test. The second phase involved statistical analysis using generalized linear models, with Poisson regression, log-link function, and robust models utilized for large datasets. Subsequently, p-values (with the significance set to < 0.05), adjusted prevalence ratios, and their 95% confidence intervals were calculated.

The rights and dignities of the participants were respected at all times, and ethics were upheld in accordance with international research standards. Additionally, the anonymity of the donors was maintained.

## Results

Of a total of 48,567 blood donors, 32,846 (67.63%) were men and 15,721 (32.37%) were women. While 37,981 (78.48%) were replacement donors, 10,536 (21.52%) were voluntary. Only 999 (2.06%) donors were positive for infectious diseases, in proportions as follows: syphilis, 0.92%; core hepatitis, 0.72%; HBV, 0.22%; HTLV, 0.20%; HIV, 0.12%; and HCV, 0.05%.

Men comprised 79.2% (791) of the total number of infection-positive donors and the median age was 36 years (interquartile range: 28–47 years). The largest percentage of donors were single (34.6%), had a up to a primary level of education (51.5%), had donated previously (37.7%), and were replacement donors (88.5%). Table 1 shows the other descriptive characteristics of the donors.

**Table 1:** Social and educational characteristics of the Honduran Red Cross–northern region blood donors who tested positive for infectious diseases between 2014 and 2016.

Variable	n = 999	%
**Sex**		
Female	208	20.8
Male	791	79.2
**Age** (years)*	36	28–47
**Year of donation**		
2014	322	32.2
2015	333	33.3
2016	344	34.5
**Civil status**		
Single	345	34.6
Married	298	29.9
Others	355	35.5
**Educational level**		
None	35	3.5
Primary	511	51.5
High school	351	35.4
College	95	9.6
**Previously donation**	377	37.7
**Type of donation**		
Voluntary	115	11.5
Replacement	884	88.5

* Median and interquartile range.

**Fig 1:**
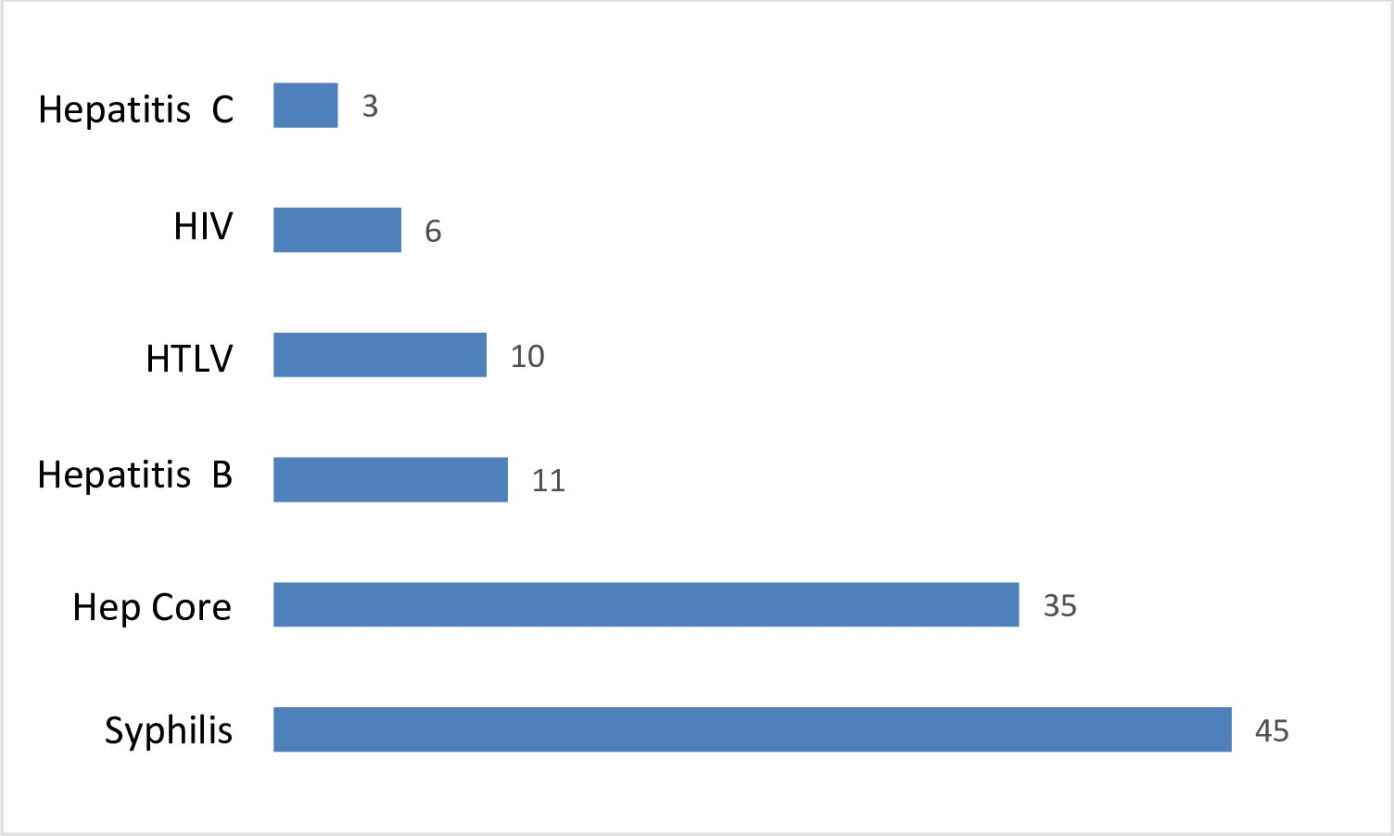
Percentages of infectious diseases among Honduran Red Cross–northern region blood donors who tested positive for infectious diseases between 2014 and 2016.

The proportions of participants who were positive for syphilis, core hepatitis, HBV, HTLV, HIV, and HCV infections were 45% (447/999), 35% (348/999), 11% (105/999), 10% (97/999), 6% (59/999), and 3% (24/999), respectively (Fig 1).

The results of the bivariate analysis showed that sex was associated with positivity to core hepatitis (p < 0.001), HIV (p = 0.024), syphilis (p = 0.002), and HTLV (p = 0.010) infection. Additionally, a correlation was observed between age and positivity for HBV (p < 0.001), HCV (p = 0.016), HIV (p < 0.001), syphilis (p < 0.001), and HTLV (p < 0.001) infections. Meanwhile, a high educational level was associated with positivity for core hepatitis (p = 0.002) and HTLV (p = 0.020) infections. A relationship was also observed between a history of donation and positivity for core hepatitis (p < 0.001), HBV (p = 0.001), HCV (p = 0.042), HIV (p = 0.001), syphilis (p < 0.001), and HTLV (p < 0.001) infections. Moreover, replacement donations were associated with positivity to core hepatitis (p = 0.049) and HTLV (p < 0.001) infections (Table 2).

**Table 2:** Bivariate analysis of the infectious diseases based on the social and educational characteristics of Honduran Red Cross–northern region blood donors who tested positive for infections between 2014 and 2016.

Variable	P-values (n = 999)					
	CoreHep (n = 348)	Hep. B (n = 105)	Hep. C (n = 24)	HIV (n = 59)	Syphilis (n = 447)	HTLV (n = 97)
**Sex Female**	<0.001	0.189	0.610	0.024	0.002	0.010
**Age** (years)*	0.917	<0.001	0.016	<0.001	<0.001	<0.001
**Civil status**						
Others	Comparison group					
Single	0.842	0.698	0.323	N.C.	0.727	0.308
Married	0.178	0.701	0.172	N.C.	0.975	0.099
**Educational level**						
None	Comparison group					
Primary	0.935	0.565	0.783	N.C.	0.726	0.895
High school	0.237	0.780	0.917	N.C.	0.704	0.430
College	0.002	0.883	0.930	N.C.	0.255	0.020
**Previously donation**	<0.001	0.001	0.042	0.001	<0.001	<0.001
**Replacement donation**	0.049	0.767	0.153	0.930	0.158	<0.001

P-values were calculated with generalized linear models, Poisson regression, log-link function and robust models utilized for large datasets. N.C.: Does not converge due to small sample.

* Median and interquartile range.

Meanwhile, the results of the multivariate analysis demonstrated that the number of women who tested positive for HIV infection was lower than that of men (p = 0.006). Furthermore, older participants were more likely to be positive for core hepatitis (p = 0.029) and syphilis (p < 0.001) infections but were less likely to be positive for HBV (p < 0.001), HCV (p = 0.027), HIV (p < 0.001), and HTLV (p < 0.001) infections than were younger participants. Previous blood donation increased the likelihood of positivity for core hepatitis (p < 0.001) infection but decreased the likelihood of positivity for HBV (p = 0.001), HIV (p < 0.001), and HTLV (p = 0.001) infections. In contrast, replacement donations increased the likelihood of positivity for core hepatitis (p = 0.003) infection but decreased the likelihood for positivity for HTLV (p = 0.001) infection (Table 3).

**Table 3:** Multivariate analysis of the infectious diseases based on the social and educational characteristics of the Honduran Red Cross–northern region blood donors who tested positive for infections between 2014 and 2016.

Variable	Prevalence ratio (95% CI) and P-values (n = 999)					
	CoreHep (n = 348)	Hep. B (n = 105)	Hep. C (n = 24)	HIV (n = 59)	Syphilis (n = 447)	HTLV (n = 97)
**Sex** **Female**	0.9(0.7–1.1) 0.400	1.1(0.7–1.6) 0.710	0.9(0.4–2.5) 0.951	0.2(0.1–0.6) 0.006	1.1(0.9–1.2) 0.085	1.3(0.9–1.9) 0.156
**Age** (years)*	1.0(1.0–1.0) 0.029	0.9(0.9–0.9) <0.001	0.9(0.9–0.9) 0.027	0.9(0.9–0.9) <0.001	1.0(1.0–1.0) <0.001	0.9(0.9–0.9) <0.001
**Previous donation**	6.4(5.1–8.1) <0.001	0.5(0.3–0.7) 0.001	0.3(0.1–1.0) 0.052	0.3(0.1–0.6) <0.001	0.2(0.2–0.3) <0.001	0.4(0.3–0.7) 0.001
**Replacement donation**	1.5(1.1–1.9) 0.003	1.0(0.6–1.8) 0.875	0.5(0.2–1.5) 0.243	1.1(0.5–2.3) 0.751	1.0(0.8–1.3) 0.646	0.5(0.3–0.8) 0.001

Prevalence ratios (95% confidence interval) and P-values were calculated with generalized linear models, Poisson regression, log-link function and robust models utilized for large datasets.

* Median and interquartile range.

## Discussion

Close to half of the blood donors who tested positive for infectious diseases had syphilis. The high prevalence of syphilis in the present study was consistent with that reported by the Plan Estratégico Nacional de Lucha Contra al VIH/SIDA PENSIDA (National Strategic Plan to Fight HIV/AIDS, 2015), which also revealed that sexually transmitted infections continue to be a public health burden. The overall national prevalence of syphilis varied from 1.0% in 2008 to 2.5% in 2012. Men who have sexual relations with men have the highest risk of acquiring this infection [12]. The high percentage of blood donors positive for syphilis infection in this study was consistent with the findings of a study in another health institution (the blood bank at the University School Hospital in Tegucigalpa) in 2014, which reported that 20% of blood donors positive for infectious diseases had syphilis [5]. Significant changes in the prevalence of syphilis should alert healthcare providers, given that the rise may be attributed to changes in the prevalence trends of other related diseases or in specific populations. Further studies are warranted to validate this finding so that necessary interventions can be implemented to prevent syphilis from becoming a global health problem.

In this study, a high percentage of the infected blood donors were positive for core hepatitis and HBV infections, a finding consistent with the that of a previous study in Tegucigalpa, which reported a high prevalence of core hepatitis infection among its participants [5]. Although different prevalence rates have been reported in other studies performed in a similar population, HCV (35%) was reported to be more common than HBV (10%) among the donors of blood banks in Mexico [13,14], highlighting an additional risk of which healthcare professionals should be aware. Additionally, living in rural areas was significantly related to an increased risk of HBV infection [15]. Hence, programs on the prevention of sexually transmitted diseases (especially among young people) and on work-related accidents in specific populations, such as healthcare professionals and those working in rural areas, should be intensified as hepatitis is most commonly transmitted through exposure to infected blood, semen, and other body fluids.

HTLV infection ranked fourth among the infections with the highest prevalence in the present study. This finding was consistent with that of a study conducted in the same population at the beginning of the present century [10], and the high prevalence of this infection should be considered when processing blood donations. Another study of blood donors from Guatemala showed not only that HTLV infection is common but that it also commonly affects men between 18 and 29 years of age [16]. However, another study in Colombia reported that women are more commonly infected with HTLV and that it is more prevalent among younger individuals [17]. Considering the high prevalence of HTLV among younger individuals, promotion and prevention campaigns should focus on this population.

Additionally, the findings of our study showed a lower prevalence of HIV infection in women, a finding similar to prevalence rates of approximately 68% and 71% among men in Brazil [18] and Peru [19], respectively. Similar findings of a higher prevalence in men have also been reported in other parts of Latin America, as well as North America and Europe. Men who have sexual relationships with men have reportedly have the highest risk of infection with HIV [17, 20]. However, this finding is controversial, as reports have also shown that women account for the highest proportion of patients infected with HIV globally, representing approximately 51% of the HIV-positive population [21]. This change in the demographics of HIV infection is mostly attributed to various anatomical, histological, and social factors [22], as well as to sexual practices among various groups. A 2010 report in Nicaragua indicated that a large percentage of homosexual men also had sex with women [23]. Based on this report, the incidence of HIV infection among men who have sexual relationships with men is low, whereas that among heterosexuals is high [24]. This finding should be considered in the analysis of those groups who are at increased risk of infection so that additional efforts can be made to address the changes in the demographics of this disease.

Furthermore, the present study also observed an association between age and infection positivity. Specifically, older donors had increased positivity rates for syphilis or core hepatitis infections, whereas younger participants were more likely to be positive for the other evaluated diseases than were their older counterparts. This finding is consistent with those of previous studies that reported that the majority of participants positive for viral disease infections were young adults or youths (especially among those 30 ± 10 years of age), most of whom were students, worked in the military, or were engaged in other professions common in this age group [25,26]. Therefore, younger donors should receive intensive counseling on topics related to sexual practices. Additionally, sex education programs should also be provided in schools and universities.

Meanwhile, a history of previous blood donation was associated with a decreased likelihood of positivity to almost all of the infectious diseases. This finding may be attributed to the fact that patients who donate regularly are screened for any diseases and, thus, are aware that they do not have these infections. Hence, obtaining information on the history of blood donations remains a suitable means of screening for such diseases. Global initiatives have been established to ensure universal access to safe blood. One of these initiatives is the creation of a system where unpaid regular voluntary donors are preferred over family or replacement donors. This initiative is valuable because donors develop a sense of responsibility toward their community and strive to remain healthy to be able to continue donating safe blood [27]. One of the overall objectives of the World Health Organization is that all countries will obtain their blood supply from volunteer rather than replacement donors by the year 2020 [7]. Each country and health institution should develop strategies to achieve this goal. Given that the type of donor is associated with positivity to two of the evaluated infectious diseases, encouraging a culture of voluntary blood donations and provision of education programs on healthy lifestyle and sexual practices are important in ensuring the safety of blood donations.

The results of the present study are limited by selection bias, given that a census sample was taken from a single region of Honduras from among donors who were positive for an infection. Thus, the findings cannot be generalized to all people in Honduras. However, Tegucigalpa is one of the most important regions in the country and its inhabitants share similar characteristics with those of other populations in the country and surrounding places. The results of this study should be considered preliminary findings and further research involving a large population and multiple settings is warranted.

## Conclusions

In the present study, nearly half of the infected blood donors were positive for syphilis and a high number of participants were positive for hepatitis B, core hepatitis, and HTLV infection. Women had a lower prevalence of HIV infection than that in men and an association between age and positivity for all infectious diseases was also observed. The high prevalence of infectious diseases in Honduras demonstrates the need for the ongoing monitoring of the blood supply to prevent infected blood from being provided for transfusions. Furthermore, education efforts should be made through the creation of a prevention program to educate the Honduran population, especially younger individuals, about transfusion-transmissible infections.
